# Relocating Glyceryl Trinitrate as an Anti-Virulence Agent against *Pseudomonas aeruginosa* and *Serratia marcescens*: Insights from Molecular and In Vivo Investigations

**DOI:** 10.3390/microorganisms11102420

**Published:** 2023-09-28

**Authors:** Shaimaa I. Nazeih, Mohamed A. M. Ali, Alyaa S. Abdel Halim, Hanan Al-Lawati, Hisham A. Abbas, Mohammed Al-Zharani, Fehmi Boufahja, Mashael A. Alghamdi, Wael A. H. Hegazy, Noura M. Seleem

**Affiliations:** 1 Department of Microbiology and Immunology, Faculty of Pharmacy, Zagazig University, Zagazig 44519, Egypt; shaimaa_nazeih@yahoo.com (S.I.N.); hishamabbas2008@gmail.com (H.A.A.); noura.seleem@gmail.com (N.M.S.); 2 Department of Biology, College of Science, Imam Mohammad Ibn Saud Islamic University (IMSIU), Riyadh 11623, Saudi Arabia; mamzaid@imamu.edu.sa (M.A.M.A.); faboufahja@imamu.edu.sa (F.B.); 3 Department of Biochemistry, Faculty of Science, Ain Shams University, Abbassia, Cairo 11566, Egypt; alyaa.saher@sci.asu.edu.eg; 4 Pharmacy Program, Department of Pharmaceutics, Oman College of Health Sciences, Muscat 113, Oman; allawati@ualberta.ca; 5 Department of Chemistry, Imam Mohammad Ibn Saud Islamic University (IMSIU), Riyadh 11623, Saudi Arabia; mabalghamdi@imamu.edu.sa; 6 Pharmacy Program, Department of Pharmaceutical Sciences, Oman College of Health Sciences, Muscat 113, Oman

**Keywords:** glyceryl trinitrate, bacterial virulence inhibition, *Pseudomonas aeruginosa*, *Serratia marcescens*, quorum sensing

## Abstract

The problem of antibiotic resistance is a global critical public health concern. In light of the threat of returning to the pre-antibiotic era, new alternative approaches are required such as quorum-sensing (QS) disruption and virulence inhibition, both of which apply no discernible selective pressure on bacteria, therefore mitigating the potential for the development of resistant strains. Bearing in mind the significant role of QS in orchestrating bacterial virulence, disrupting QS becomes essential for effectively diminishing bacterial virulence. This study aimed to assess the potential use of sub-inhibitory concentration (0.25 mg/mL) of glyceryl trinitrate (GTN) to inhibit virulence in *Serratia marcescens* and *Pseudomonas aeruginosa.* GTN could decrease the expression of virulence genes in both tested bacteria in a significant manner. Histopathological study revealed the ability of GTN to alleviate the congestion in hepatic and renal tissues of infected mice and to reduce bacterial and leukocyte infiltration. This study recommends the use of topical GTN to treat topical infection caused by *P. aeruginosa* and *S. marcescens* in combination with antibiotics.

## 1. Introduction

Bacterial resistance poses a significant challenge to public health, stemming from the ongoing development of antibiotic resistance by bacteria and the reduced introduction of new antibiotics to the market. About 700,000 annual deaths occur globally due to infections with resistant bacteria. This number is expected to reach 10 million annual deaths by 2050. Resistant bacterial infections will be the leading cause of death by 2050 [1,2,3]. The issue of resistance development is amplified and has become more pressing, especially when dealing with highly virulent bacteria like *Pseudomonas aeruginosa*.

*P. aeruginosa* belongs to the group of ESKAPE pathogens, which encompasses the foremost six bacteria responsible for healthcare-associated infections with multidrug-resistant traits [4,5,6]. Being an opportunistic bacterium, *P. aeruginosa* has the potential to induce various infection types, particularly in individuals with compromised immune systems [4,7]. These infections encompass pneumonia, urinary tract infections, surgical site infections, and bloodstream infections [8,9,10]. Additionally, it can trigger burn and wound infections, as well as ocular infections [11,12]. *P. aeruginosa* has an exceptional resistance to antibiotics, either intrinsic or acquired [13]. Furthermore, it has a powerful arsenal of virulence factors that help adhere, colonize, and invade host tissue [14,15]. This arsenal includes protease, elastase, phospholipase, hemolysin enzymes, biofilm formation, and pyocyanin pigment [16,17]. Also, the ability of this bacterium to swim, swarm, and twitch enables it to attach to host tissue to initiate biofilm formation [12,18,19]. Biofilm formation capability adds to the virulence and antibiotic resistance of *P. aeruginosa* because of the impermeability of the biofilm matrix to antibiotics and the host immune cells [20,21].

Like *P. aeruginosa*, *Serratia marcescens* shows resistance to different groups of antibiotics, including beta-lactams, fluoroquinolones, and aminoglycosides [22,23,24]. *S. marcescens* is one of the Gram-negative bacilli and a member of the family *Enterobacteriaceae*. It is one of the important emerging healthcare-associated bacteria as it ranks seventh among bacteria that cause nosocomial pneumonia and tenth among bacteria that cause nosocomial bloodstream infections [25,26,27]. It also causes about 11% of burn-related surgical wound infections. Endocarditis, urinary tract infection, and osteomyelitis are also on the list of infections that can be caused by *S. marcescens* [28,29,30]. To cause infection, *S. marcescens* employs its arsenal of virulence factors that include hemolysin, protease, nuclease, chitinase, and lipase [31,32]. *S. marcescens* moves by swimming and swarming motilities and can form biofilms that increase its resistance to antibiotics [23,26]. It produces a characteristic red pigment termed prodigiosin that helps suppress immunity [33,34].

In light of the high resistance and virulence of both *P. aeruginosa* and *S. marcescens*, therapeutic options other than antibiotics are needed for these bacterial infections. These alternative therapies should not target bacterial growth because this will lead to the emergence of resistant strains [35,36]. Targeting virulence mechanisms seems to satisfy this requirement by allowing bacterial growth while concurrently impeding its virulence. This prevents the bacteria from causing infections, rendering them susceptible to elimination by the host’s immune system [37,38,39,40,41]. To inhibit the virulence mechanism in bacteria, a quorum-sensing system (QS) that controls the expression of virulence factors seems the ideal target [37,42]. QS is a system of communication between bacterial cells that works in an inducer/receptor manner [43,44]. These are known as autoinducers or signaling molecules that are secreted by bacterial cells. It is noteworthy that the concentration of these autoinducers reflects the number of bacterial cells. Autoinducers accumulate in the surrounding environment till a threshold concentration is achieved. Then, autoinducers bind to specific receptors that act as transcriptional regulators [43]. The receptors undergo conformational change and dimerization to have a high binding affinity to specific sequences on the DNA to activate the expression of virulence genes [45,46].

Acyl homoserine lactones (AHLs) are the autoinducers of many Gram-negative bacteria [47,48]. In *P. aeruginosa*, there are three main QS systems: LasI/R, RhlI/R, and PqsA/R [4,49]. LasI synthase enzyme produces 3-oxo-dodecanoyl homoserine lactone that binds to LasR when it reaches a threshold concentration to elicit changes in the expression of virulence genes encoding exo-protease, elastase, exotoxin A, and biofilm. Butanoyl homoserine lactone autoinducer is produced by RhlI synthase and binds to the RhlR receptor. The RhlI/R system regulates the production of siderophores, pyocyanin, elastases, and rhamnolipids [43,50]. In *Serratia* spp., two AHL-mediated QS systems are present, namely SwrI/R and SmaI/R [51,52]. These systems use butanoyl and hexanoyl acyl homoserine lactones that, when bound to their receptors, activate the expression of virulence genes such as prodigiosin, protease, hemolysin, biofilm formation, and swarming motility [12,26].

**Figure 1:**
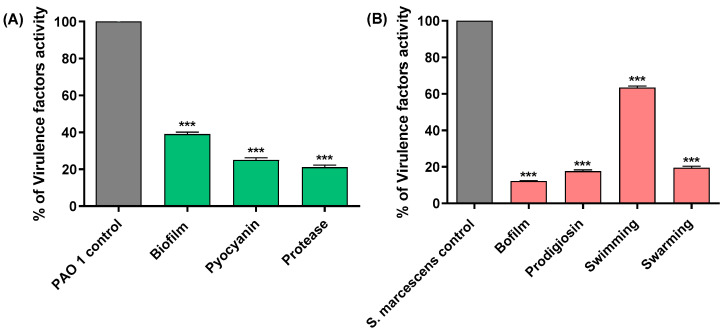
Phenotypic assessment of anti-virulence activities of GTN against (**A**) *P. aeruginosa* PAO1 and (**B**) *S. marcescens*. GTN significantly decreased the biofilm formation, swimming, and swarming motilities, and virulence factors (protease, pyocyanin, and prodigiosin) production in *P. aeruginosa* and *S. marcescens*. (*** = *p* < 0.001).

Drug repurposing of FDA-approved drugs is a good strategy that allows the screening of a large library of drugs to discover novel uses for them. This has the merits of saving the cost and time needed for the synthesis of new antibiotics. Moreover, the safety and toxicity, in addition to pharmacokinetic properties, are well known [53,54,55]. Glyceryl trinitrate (GTN) is an antihypertensive drug [56]. GTN has antimicrobial and antibiofilm activities in addition to its wound-healing effect [57]. When GTN was employed as a catheter lock solution with citrate and ethanol, it removed the biofilms of methicillin-resistant *Staphylococcus aureus*, *P. aeruginosa*, *Klebsiella pneumoniae*, *Enterococcus faecalis*, *Enterobacter* spp., *Escherichia. coli*, *Stenotrophomonas maltophilia* and *Candida albicans*. GTN ointment was approved by the FDA for treating anal fissures at a concentration of 0.4% [57,58,59]. In our previous work, the anti-virulence activities of GTN against *P. aeruginosa* [60] and *S. marcescens* [61] were phenotypically reported. GTN significantly diminished the production of virulence factors, motility, and biofilm formation in *P. aeruginosa* and *S. marcescens* (Figure 1). The present research seeks to delve deeper into the genotypic, in vivo, and in silico effects of GTN on QS-mediated virulence factors and biofilm formation. This exploration aims to uncover anti-virulence mechanisms targeting the *P. aeruginosa* PAO1 strain and a clinical *S. marcescens* isolate obtained from surgical wound infection.

## 2. Materials and Methods

### 2.1. Bacterial Strains, Media, and Chemicals

*P. aeruginosa* (PAO1) standard strain [7] and clinical isolate *S. marcescens*, isolated from an ICU patient with a surgical wound [12,23], were used in this study. All the used media were purchased from Lab M Limited (Lancashire, UK). All other chemicals used were of pharmaceutical grade. Glyceryl trinitrate was procured from POHL-Boskamp, Gmbh & Co. in Hohenlockstedt, Germany.

### 2.2. Determination of Minimum Inhibitory Concentration (MIC)

The Clinical Laboratory and Standards Institute (CLSI) Guidelines were followed to determine the MIC of GTN against the tested strains using the agar dilution method [62,63].

### 2.3. Quantification of QS-Encoding and Virulence-Encoding Genes

qRT-PCR was used to quantify the expression of QS- and virulence-encoding genes in *P. aeruginosa* and *S. marcescens*. The total RNA was extracted from fresh overnight cultures (cell density of 10^7^ CFU/mL) that were provided or not with GTN at sub-MIC (0.25 mg/mL). The Gene JET RNA Purification Kit (Thermo Scientific, Waltham, MA, USA) was used, and the isolated RNA was kept at −80 °C as described [7,64].

The QS-encoding genes in *P. aeruginosa lasI*, *lasR*, *rhlI*, *rhlR*, *pqsA*, and *pqsR* were amplified using primers that were listed [7,65]. The involved genes in *S. marcescens* virulence (*fimC*, *flhD*, *bsmB*, *rssB*, and *pigP*) were amplified using the aforementioned primers [23,66]. The relative gene expression was calculated using the comparative threshold cycle (∆∆Ct) method. The housekeeping genes *rpoD* and *rplU* were used for *P. aeruginosa* and *S. marcescen*, respectively, to normalize the relative expression level of each tested gene. The amplification procedure was conducted according to SensiFAST™ SYBR^®^ Hi-ROX OneStep Kit (Bioline, London, UK) using the StepOne Real-Time PCR system (Applied Biosystem, Waltham, MA, USA).

### 2.4. Histopathological Study

To study the effect of glyceryl trinitrate on the pathogenesis of *P. aeruginosa* and *S. marcescens*, 30 albino mice aged three weeks were optimally housed in the animal house at the Faculty of Pharmacy, Zagazig University. The mice were grouped into six groups, each of five mice. The first and second groups were intraperitoneally injected with GTN-treated *P. aeruginosa* and *S. marcescens*, respectively. The third and fourth groups were injected with untreated *P. aeruginosa* and *S. marcescens*, respectively. Two negative control groups (the fifth and sixth groups) were used. The fifth one was injected with phosphate-buffered saline and the sixth group was untreated. The mice were allowed to remain undisturbed for a period of 5 days, after which they were humanely euthanized through cervical dislocation. The liver and kidney tissues were removed from the mice and washed with normal saline before fixation in 10% formalin for histopathological investigation. The tissues were dehydrated using ethanol in increasing concentrations before clearance in xylol, impregnation, and embedding in paraffin wax. Subsequently, tissue sections, 5 μm in thickness, were sliced using a rotatory microtome. These sections were then stained with hematoxylin and eosin (H&E) stain (magnification ×200), and examination was performed using a Leica DM750 HD digital microscope from Mannheim, Germany [7,67]. 

### 2.5. Virtual Evaluation of the Binding Affinity to QS Synthetase and Receptors in P. aeruginosa and S. marcescens

Molecular docking analysis of GTN and the co-crystallized ligands was carried out with *P. aeruginosa* QS receptors, namely QscR, RhlR, LasR, and PqsR, and autoinducer synthetases PqsA and LasI. Furthermore, GTN was also docked with *S. marcescens* SmaR-like receptor [66]. The crystal structure of the receptors in complex with their ligands was retrieved from the Protein Data Bank (PDB).

### 2.6. Statistical Assessment

The experiments were carried out in triplicate, and the outcomes were averaged. The results were presented as the mean ± standard deviation. To assess statistical significance, the student t-test was employed, with significance determined when *p* < 0.05.

## 3. Results

### 3.1. The Antibacterial Activity of GTN

GTN inhibited the growth of both *P. aeruginosa* and *S. marcescens* at concentrations higher than 1 mg/mL. As a result, a concentration of 0.25 mg/mL that is equal to ¼ MIC of GTN or lower was selected for further examination of the anti-virulence activities of GTN. It is worth mentioning that the effect of GTN at the selected concentration (0.25 mg/mL) does not influence the growth of tested strains as was shown [60,61].

### 3.2. GTN Down-Regulates the Expression of P. aeruginosa QS Genes

**Figure 2:**
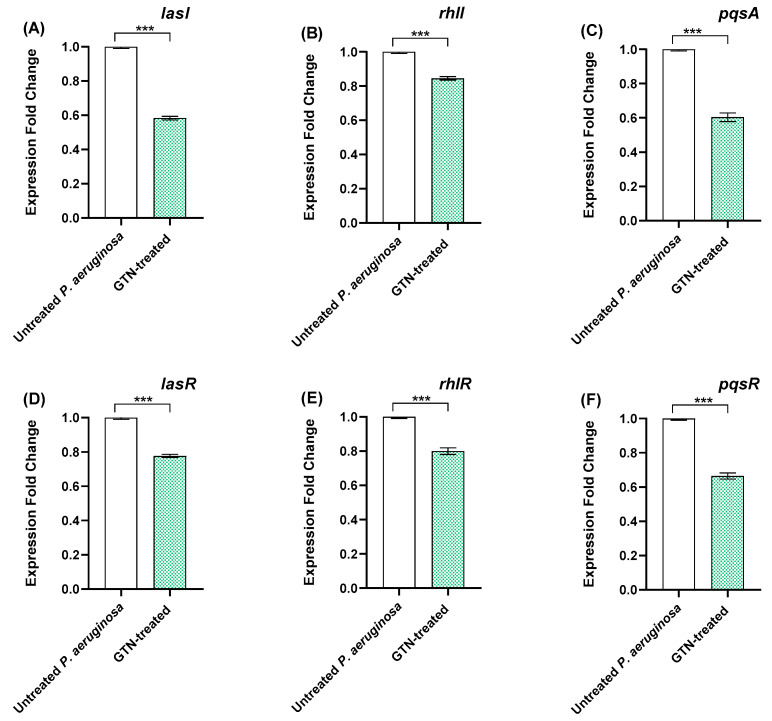
Sub-MICs of GTN decreased the *P. aeruginosa* QS gene expressions. RT-qPCR revealed decreased expression of QS-encoding genes (**A**) *lasI*, (**B**) *rhlI*, (**C**) *pqsA*, (**D**) *lasR*, (**E**) *rhlR*, and (**F**) *pqsR* in treated *P. aeruginosa* compared to untreated control. Data shown represent the mean ± SE from three experiments (***: *p*-value < 0.001).

Using qRT-PCR, the down-regulation of QS genes in *P. aeruginosa* by GTN was investigated. (Figure 2). GTN caused remarkable down-regulation of all tested genes. As compared to control untreated PAO1. The expression levels of lasI, rhlI, pqsA, lasR, rhlR, and pqsR decreased by 41.67, 15.38, 40, 20.43, 20, and 33.33%, respectively. These findings indicate a significant down-regulation effect of GTN at sub-MIC on all the QS-encoding genes in *P. aeruginosa*.

### 3.3. GTN Down-Regulates the Expression of S. marcescens Virulence Genes

**Figure 3:**
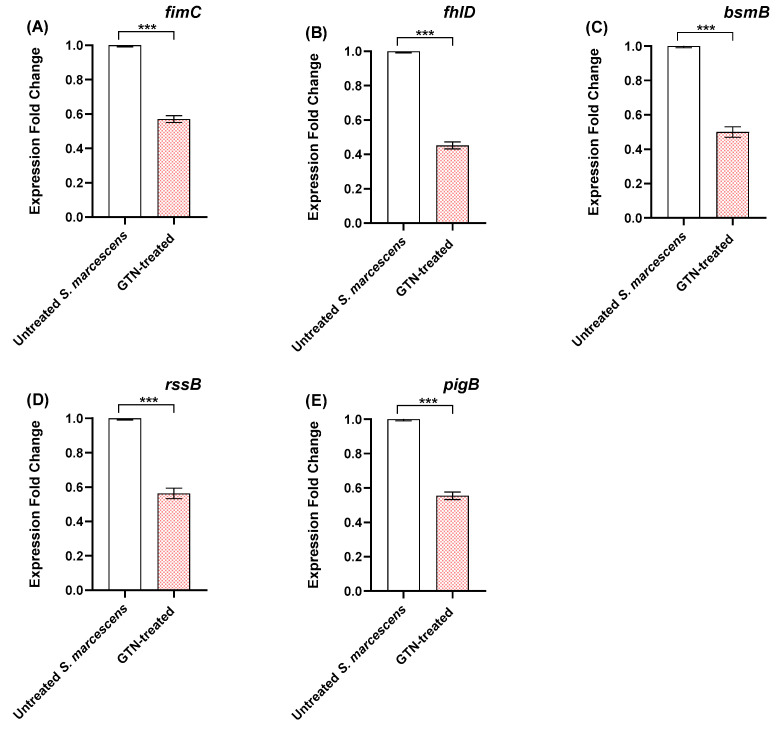
Sub-MICs of GTN decreased the *S. marcescens* virulence gene expressions. RT-qPCR revealed decreased expression of (**A**) *fimC*, (**B**) *fhlD*, (**C**) *bsmB*, (**D**) *rssB*, and (**E**) *pigB*, in treated *S. marcescens* compared to untreated control. Data shown represent the mean ± SE from three experiments (***: *p*-value < 0.001).

The qRT-PCR revealed that GTN at sub-MIC significantly decreased the relative expressions of virulence factors regulating genes in *S. marcescens* (Figure 3). GTN significantly decreased adhesion involved genes *fimC* and *bsmB* genes 42.86 and 50%, respectively. GTN down-regulated the expression of the genes *flhD* and *rssB* that are involved in *S. marcescens* motility by 54.55 and 43.75%, respectively. Moreover, the expression of *S. marcescens* virulent pigment prodigiosin encoding gene *pigB* was down-regulated by 43.44%.

### 3.4. GTN Acquires Affinity to Hinder QS Systems in P. aeruginosa and S. marcescens

Detailed docking results, including binding free energy, RMSD values, and type of interactions, are summarized in Table 1.

**Figure 4:**
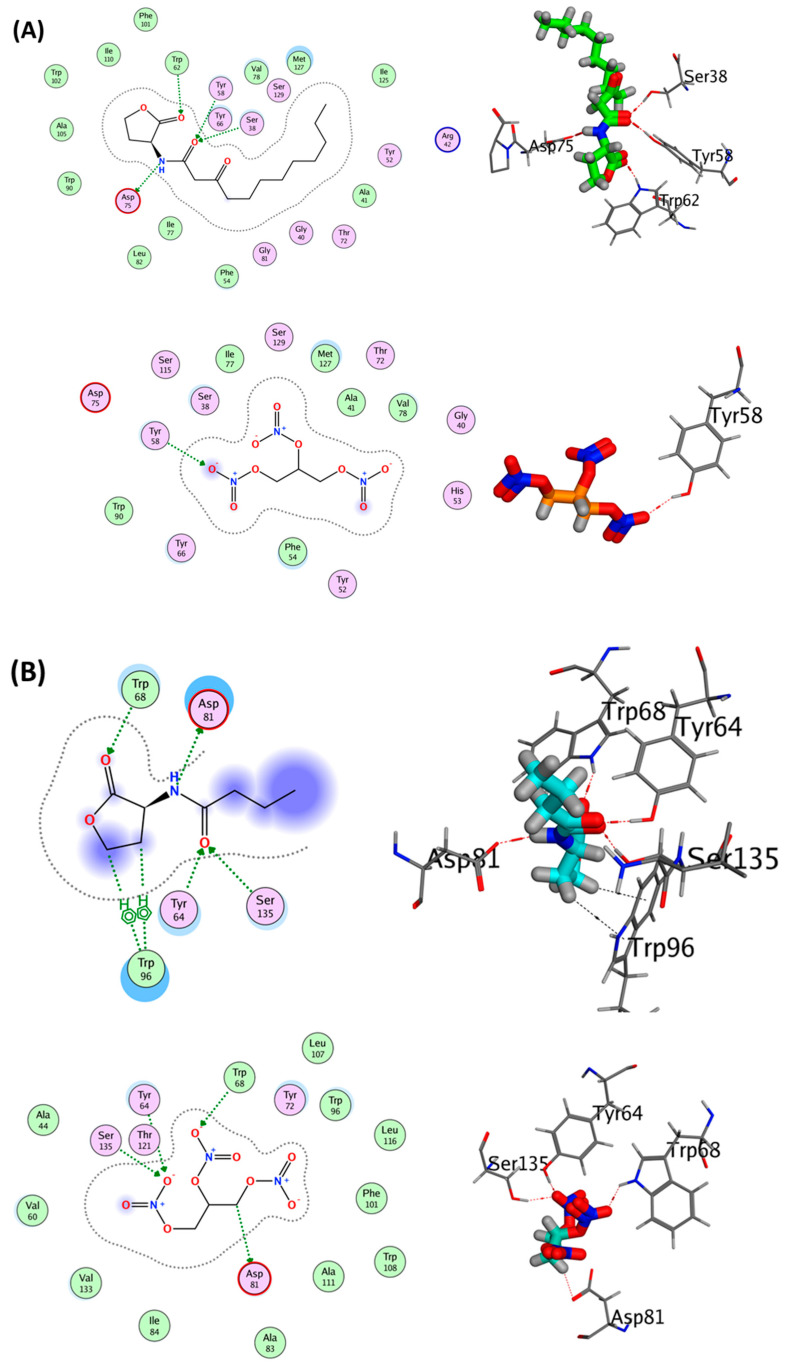

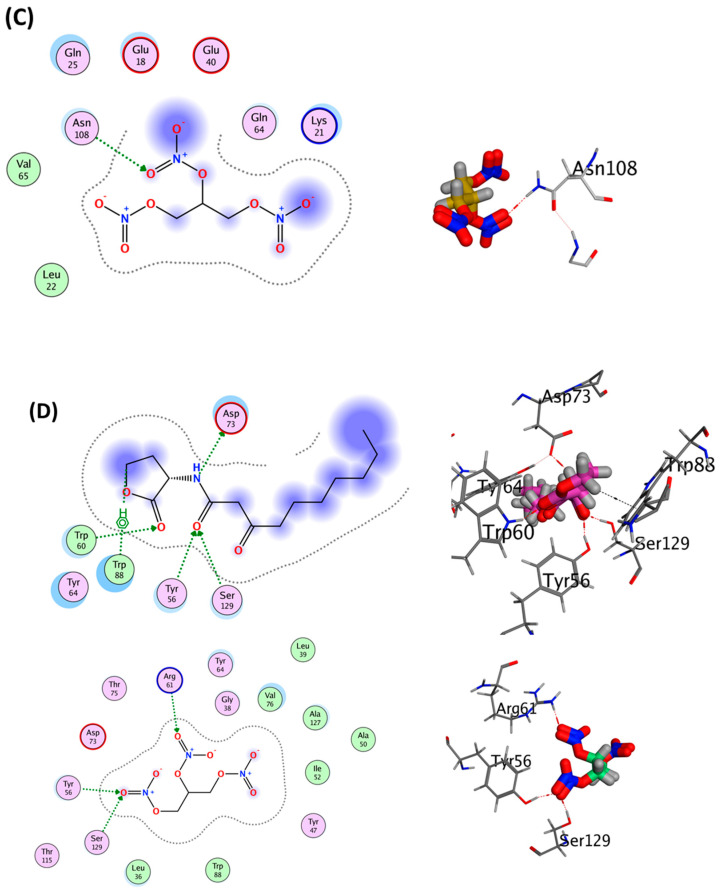

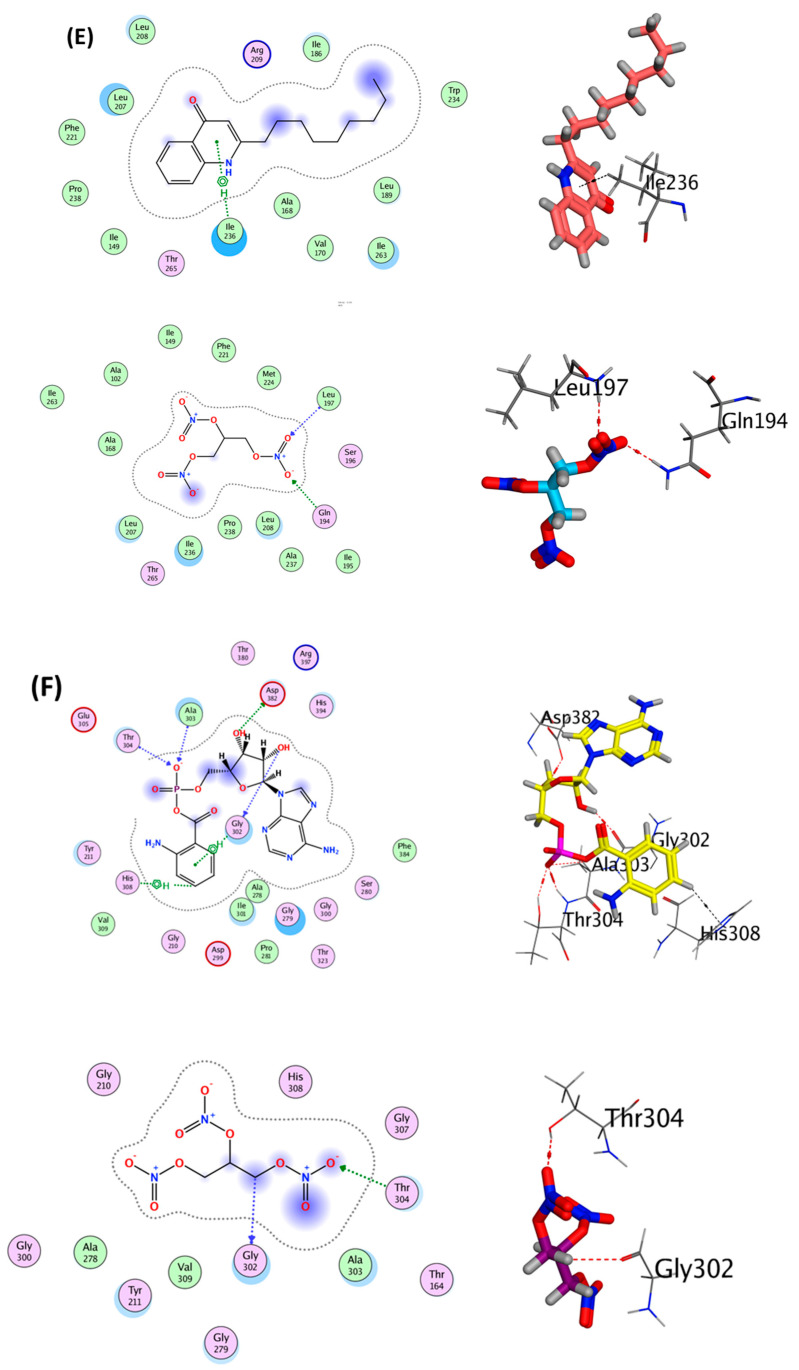

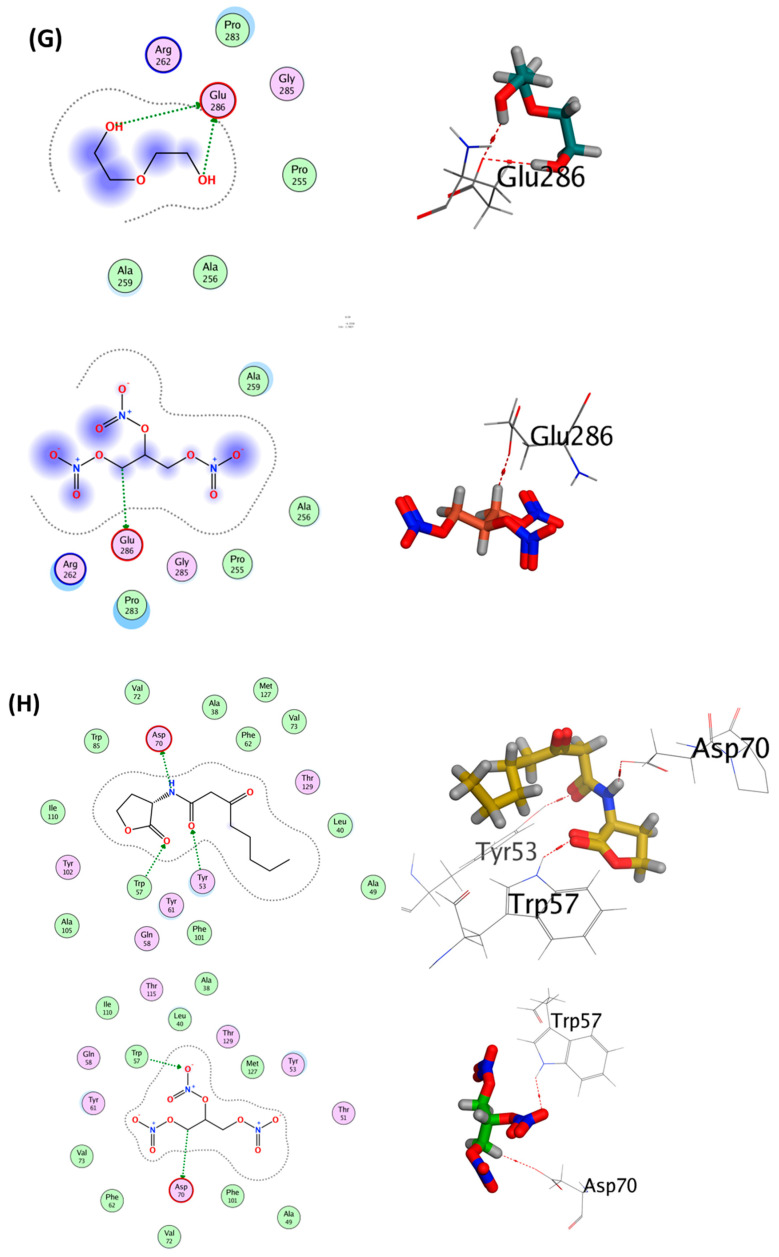
The virtual interactions between GTN to *P. aeruginosa* or *S. marcescens* QS targets. The GTN showed binding affinity to *P. aeruginosa* QS targets (**A**) QscR, (**B**) RhlR, (**C**) LasI, (**D**) LasR, (**E**) PqsR, (**F**) PqsA (3UK ligand), and (**G**) PqsA (PEG ligand) in addition to *S. marcescens* SmaR (**H**). The upper figures in each target represent the natural ligand while the lower figures represent the GTN interactions to each target.

As for QscR (PDB ID: 3SZT), GTN possesses a binding score of −7.06 kcal/mol with a single interaction involving one hydrogen bond with Tyr58, while the co-crystallized ligand OHN exhibited a binding score of −10.08 kcal/mol with four major interactions, including four hydrogen bonds with the amino acids Ser38, Tyr58, Trp62, and Asp75 at 2.89, 2.89, 3.21, and 2.82 Å, respectively (Figure 4A).

The co-crystallized ligand HL4 of RhlR (PDB ID: 8B4A) demonstrated a binding score of −6.55 kcal/mol with six interactions, including four hydrogen bonds with the amino acids Tyr64, Trp68, Asp81, and Ser135 at 2.73, 2.96, 2.89, and 2.83 Å, respectively, as well as two H-π interactions with Trp96 at 3.80 and 4.21 Å, respectively. Interestingly, GTN revealed a binding score comparable to that of the co-crystallized ligand (−6.54 kcal/mol) with the same hydrogen-bonding interactions observed with the co-crystallized ligand (Tyr64, Trp68, Asp81, and Ser135) (Figure 4B).

Two ions have been identified as LasI-type (PDB ID: 1RO5) ligands, namely sulfate ion (SO_4_) and zinc ion (Zn^2+^). Sulfate interactions include five hydrogen bonds: three with the amino acid Gly35, one with Asp37, and another one with Val38. On the other hand, zinc is involved in metal coordination with His3. GTN exhibited a binding score of −4.85 kcal/mol with a single hydrogen-bonding interaction involving Asn108 at 3.03 Å (Figure 4C).

Regarding LasR (PDB ID: 6MVN), its co-crystallized ligand 3M5 demonstrated a binding score of −10.63 kcal/mol with five interactions, including four hydrogen bonds with the amino acids Tyr56, Trp60, Asp73, and Ser129 at 2.72, 3.09, 2.81, and 2.63 Å, respectively, as well as a H-π interaction with Trp88 at 4.03 Å. GTN, on the other hand, revealed a binding score of −6.7 kcal/mol with three hydrogen-bonding interactions with the amino acids Tyr56, Arg61, and Ser129 at 2.82, 2.94, and 2.94 Å, respectively (Figure 4D).

NNQ, the co-crystallized ligand of PqsR (PDB ID: 4JVD), demonstrated a binding score of −6.5 kcal/mol with a single π-H interaction with Ile236 at 3.78 Å. It is worth noting that GTN exhibited a binding score comparable to that noted with NNQ (−6.3 kcal/mol) but with different interactions, including two hydrogen bonds with the amino acids Gln194 and Leu197 at 2.98 and 3.11 Å, respectively (Figure 4E).

3UK and PEG have been identified as ligands for PqsA (PDB ID: 5OE5). The co-crystallized ligand 3UK demonstrated a binding score of −10.5 kcal/mol with seven interactions, including five hydrogen bonds with the amino acids Gly302, Ala303, Thr304, Thr304, and Asp382 at 2.60, 3.28, 2.74, 3.00, and 2.79 Å, respectively, as well as one H-π interaction with His308 at 3.71 Å, and one π-H interaction with Gly302 at 3.74 Å. In addition to the interactions mentioned above, 3UK is involved in water-mediated interactions with Gln192, Thr194, Gly206, Gly207, Arg272, and Arg297. GTN revealed a binding score of −5.88 kcal/mol with two hydrogen-bonding interactions, involving the amino acids Gly302 and Thr304 at 3.23 and 3.01 Å, respectively (Figure 4F). The co-crystallized ligand PEG, on the other hand, exhibited a binding score of −3.71 kcal/mol with two hydrogen-bonding interactions involving the amino acid Glu286 at 3.12 and 3.26 Å. GTN demonstrated a binding score of −4.33 kcal/mol with a single hydrogen-bonding interaction, involving the amino acid Glu286 at 3.34 Å (Figure 4G).

Concerning *S. marcescens* SmaR, nitroglycerin possesses a binding score of −7.1 kcal/mol with two interactions involving two hydrogen bonds with Trp57 and Asp70, while the co-crystallized ligand LAE exhibited a binding score of −9.55 kcal/mol with three major interactions, including three hydrogen bonds with the amino acids Tyr53, Trp57 and Asp70 at 2.8, 2.98, and 2.96 Å, respectively (Figure 4H).

### 3.5. Alleviation of Histopathological Changes in Hepatic and Renal Tissues by GTN

**Figure 5:**
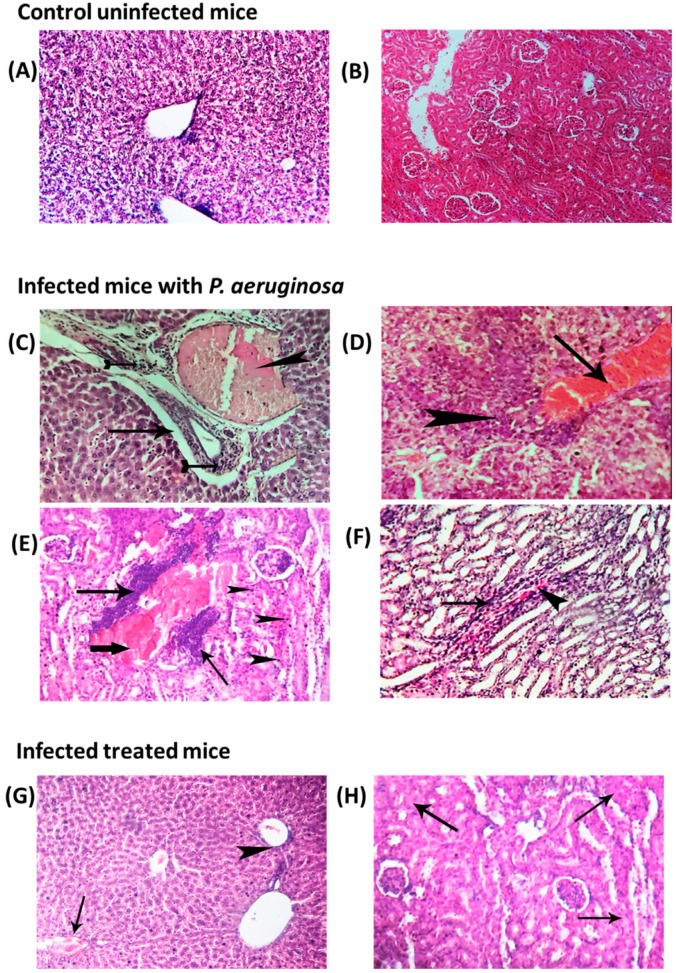
Effect of GTN on pathogenesis of *P. aeruginosa*. Photomicrographs of H&E-stained sections were captured from (**A**) liver and (**B**) kidney of uninfected mice groups displaying a typical tissue structure and normal cellular characteristics. Photomicrograph of liver sections isolated from infected mice displaying severe congestion of hepatic blood vessels (arrowhead) with perivascular and periductal colonization of microorganism with leucocytic infiltration (tailed arrow) beside periductal and peri-vascular edema (arrow) (**C**). Furthermore, intense congestion of liver blood vessels (arrow) with perivascular necrotic area infiltrated with a few lymphocytes (arrowhead) (**D**). Photomicrograph of kidney sections isolated from infected mice displaying diffuse severe congestion of renal blood vessels (thick arrow) with perivascular colonization of microorganism and inflammatory cellular infiltration (arrows) and focal renal epithelium pyknosis (arrowhead) (**E**), and intratubular hemorrhage (arrowhead) with leucocytic cells infiltration (arrow) (**F**). On the other hand, the photomicrograph of sections captured from the GTN-treated mice group showed mild congestion of hepatic blood vessels (arrow) with mild perivascular infiltration of microorganism rods and inflammatory cellular infiltration (arrowhead) (**G**), and normal renal medulla, except for focal cloudy swelling in a few renal tubules (arrow) (**H**).

**Figure 6:**
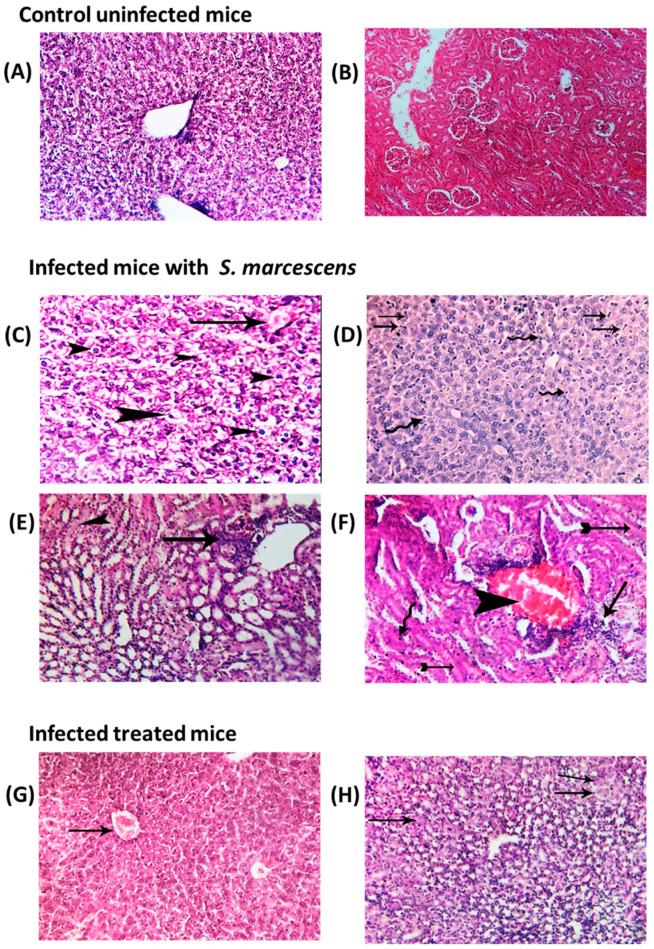
Effect of GTN on the pathogenesis of *S. marcescens*. Photomicrographs of H&E-stained sections were captured from (**A**) liver and (**B**) kidney of uninfected mice groups displaying typical tissue structure and normal cellular characteristics. Photomicrograph of liver sections isolated from infected mice displaying congestion of blood vessels (arrow) with diffuse hydropic degeneration of hepatocytes (arrow) (**C**). Additionally, diffuse spreading of microorganisms in the hepatic parenchyma (arrows) with diffuse infiltration of von Kupffer cells (arrow) (**D**). Photomicrograph of kidney sections isolated from infected mice displaying (**E**) perivascular colonization of the microorganism beside inflammatory cell infiltration (arrow) in addition to cloudy swelling of some renal tubules (arrowhead) and (**F**) perivascular colonization of microorganism beside inflammatory cell infiltration (arrow) with severe congestion (arrowhead) in addition to cloudy swelling of some renal tubules (tailed arrow) and focal renal epithelium pyknosis (curved arrow). In contrast, the photomicrograph of sections captured from the GTN-treated mice group showed mild congestion of hepatic blood vessels (arrow) with apparently normal hepatic parenchyma (**G**), and mild degenerative changes represented in the cloudy swelling of some renal tubules (arrow) (**H**).

To investigate the GTN effect on alleviating the pathogenesis of *P. aeruginosa* and *S. marcescens*, the hepatic and renal tissues of uninfected mice, mice infected with untreated *P. aeruginosa* (Figure 5) or *S. marcescens* (Figure 6), and treated infected mice were photomicrographed. Normal tissue architecture and cellular details were found in the liver or kidney of negative control groups (Figure 5A,B and Figure 6A,B). Intense hepatic blood-vessel congestion, accompanied by leukocyte infiltration, perivascular edema, or necrotic area infiltration, was found in the hepatic tissue of the untreated mice infected with *P. aeruginosa* (Figure 5C,D). Moreover, diffuse severe congestion of renal blood vessels with inflammatory cellular infiltration and inter-tubular hemorrhage was shown in the renal tissue of the untreated mice infected with *P. aeruginosa* (Figure 5E,F). On the contrary, mild congestion of hepatic blood vessels and normal renal medulla except for focal cloudy swelling in a few renal tubules were found in the tissue of the treated infected mice (Figure 5G,H). These results demonstrate the remarkable alleviation of *P. aeruginosa* pathogenesis by GTN.

Regarding the groups comprising treated or untreated mice infected with *S. marcescens*, congested hepatic blood vessels were also noticed with diffuse hydropic degeneration of hepatocytes and diffuse infiltration of von Kupffer cells (Figure 6C,D). In the liver tissues treated with GTN, milder congestion of hepatic blood vessels was found with normal hepatic parenchyma (Figure 6F). The kidney tissues of infected untreated mice showed inflammatory cell infiltration with severe congestion, cloudy swelling, and focal renal epithelium pyknosis (Figure 6E,F). On the other hand, some degenerative changes represented by cloudy swelling of some renal tubules were noticed in the treated infected mice (Figure 6G,H).

## 4. Discussion

The merit of anti-virulence therapy lies in its ability to reduce the likelihood of resistance emergence, as it does not exert pressure on bacterial growth [68,69]. Instead, it effectively diminishes bacterial virulence. The additional benefits of the anti-virulence approach include bolstering the immune system’s capacity to eliminate pathogenic bacteria and augmenting antibiotic effectiveness when used in combination with antibiotics [70,71]. Bacterial QS serves as a mechanism through which bacterial cells coordinate the activation of virulence factors throughout the infection process. Consequently, it represents a promising focus for the development of novel anti-infective agents [4,70,72]. The QS system controls many virulence factors in both *P. aeruginosa* and *S. marcescens*, including the production of diverse virulent enzymes and pigments such as protease, hemolysin, elastase, rhamnolipids, pyocyanin, and prodigiosin pigments in addition to biofilm formation and motility [39,73,74,75]. In this study, the anti-virulence activities of GTN against *P. aeruginosa* and *S. marcescens* were investigated. Importantly, the GTN anti-virulence activities were assessed at a sub-MIC concentration of 0.25 mg/mL to exclude any influence on bacterial growth.

In our previous studies, the anti-virulence potential of GTN against the Gram-negative bacteria *P. aeruginosa* and *S. marcescens* was assessed phenotypically [60,61]. GTN at 0.25 mg/mL exerted a potent activity against the virulence factors of both bacteria. Against *S. marcescens*, GTN inhibited biofilm formation, prodigiosin pigment, swimming, and swarming motilities, and diminished protease activity [61]. Moreover, the anti-pseudomonal virulence of GTN was pronounced, biofilm was inhibited, and the production of protease and pyocyanin pigment was diminished [60].

*P. aeruginosa* possesses three distinct QS systems that play pivotal roles in regulating its virulence. These systems comprise two LuxI/LuxR-type QS mechanisms and one non-LuxI/LuxR-type system known as the *Pseudomonas* quinolone signal (PQS) system [44,76]. In the first QS system, the LuxI homolog LasI synthesizes an autoinducer called C12-homoserine lactone, which is subsequently recognized by the cytoplasmic LuxR homolog, LasR [77]. In the second QS system, RhlI is responsible for the synthesis of another autoinducer, butanoyl homoserine lactone, which, at elevated concentrations, binds to RhlR, a second LuxR homolog [78]. Additionally, there is the orphan homolog QscR, which, unlike the other LuxR homologs, lacks a partner LuxI homolog. QscR, however, binds to the autoinducers produced by LasI [49]. Furthermore, the PQS system constitutes an additional non-LuxI/LuxR QS system, synthesized by PqsA, B, C, D, and H, and is detected by the regulator PqsR, also known as MvfR [79].

The current findings proved that GTN could decrease the expression of QS-regulated virulence genes in *P. aeruginosa lasI*, *lasR*, *rhlI*, *rhlR*, *pqsA*, and *pqsR*. These findings align with our prior in vitro findings, which revealed a substantial reduction in the phenotypic virulence of *P. aeruginosa* [60]. Furthermore, a detailed in silico study was conducted to evaluate the ability of GTN to bind *P. aeruginosa* QS receptors QscR, LasR, RhlR, and PqsR and the autoinducer synthetases LasI and PqsA. The current results confirmed GTN’s binding capacity to *P. aeruginosa* QS targets, supporting the potential anti-QS activity of GTN, which, in turn, reduces its virulence.

Gram-negative QS systems are commonly LuxI/LuxR-types. In *S. marcescens*, QS is regulated basically by SmaR, a member of the LuxR family [26,76]. The SmaI/SmaR QS system oversees the expression of numerous virulence factors, including prodigiosin production, pectate lyase, cellulase, caseinase, and chitinase activities, as well as swarming motility, hemolytic activity, and biofilm formation, as elaborated by the authors of [26]. The docking results of GTN into SmaR showed the GTN binding ability hindering the *S. marcescens* QS. Furthermore, the influence of GTN on the expression of *S. marcescens* virulence-involved genes was assessed.

Fimbria or pili of the adhesive organelles of *S. marcescens* have been linked to the formation of biofilms. The type I pili, encoded by the fimABCD operon, are indispensable for the early stages of *S. marcescens* biofilm formation [80,81]. Additionally, BsmA/B transcriptional factors are required to enhance the production of type I pili in *S. marcescens* [80,82]. *S. marcescens* employs a two-component system known as RssAB, consisting of a sensor kinase and a corresponding response regulator. This system is controlled by the master swarming regulator flhDC that modulates the timing of surface migration during the early lag phase [82,83]. The flhDC regulator consists of the *flhC* and *flhD* genes, which encode the flagellar transcriptional regulators FlhC and FlhD [83,84]. Additionally, RsmA plays a crucial role within the intricate regulatory network governing swarming [85,86]. Our previous results showed the significant diminishing effect of GTN on the *S. marcescens* biofilm formation and swarming motility [61]. These follow the current results that showed the significant down-regulation effect of GTN on adhesion-responsible genes *fimC*, the encoding gene of the fimbrial A subunit, and *bsmB*, which regulates the type I fimbriae expression, and the motility-controlling genes *flhC* and *rsmA*. The production of the tripyrrole red pigment QS-controlled *S. marcescens* pigment prodigiosin is regulated by the prodigiosin biosynthetic operon, pigA-N [87]. It was observed that the presence of GTN led to a substantial decrease in the expression of the *pigB* gene, which is in agreement with our previous in vitro findings [61].

To conclude the anti-virulence activity of GTN, an in vivo study was conducted to investigate the effect on alleviating the histopathological changes due to infection with the tested bacteria by treating the bacteria with GTN before injection into the mice. Interestingly, GTN could reduce the infiltration of bacteria and leucocytes into the renal and liver tissues of mice. Moreover, it decreased the congestion of hepatic and renal veins due to infection. This gives additional proof of the ability of GTN to diminish the pathogenesis of *P. aeruginosa* and *S. marcescens* in mice models.

In a nutshell, GTN can impede the binding process between QS autoinducers and their respective receptors, leading to the inhibition of QS. Moreover, GTN demonstrated potent inhibition against several QS-controlled virulence factors. This follows the in vitro and in vivo findings that affirmed the potent GTN anti-QS and anti-virulence activity. It is worth noting that GTN is safe for topical application and it is already prescribed topically to treat anal fissures (0.4% or 4 mg/mL) [58]. However, in our study, only 0.25 mg/mL is effective as anti-virulence. This guarantees the safe application of GTN in topical infections. This suggests GTN could be combined with an adjuvant to be used in the treatment of topical infections caused by *P. aeruginosa* and *S. marcescens*.

## 5. Conclusions

GTN has demonstrated notable efficacy in decreasing the expression of various QS-encoding and QS-controlled virulence factors in *P. aeruginosa* and *S. marcescens*. It possesses the ability to bind to QS targets in *P. aeruginosa* and *S. marcescens* resulting in the disruption of QS and diminishing the virulence. These results align with both in vitro and in vivo findings, affirming GTN’s potent anti-QS and anti-virulence properties. Bearing in mind that GTN is already prescribed in the treatment of anal fissures topically, further investigations are required to apply GTN as an adjuvant to antibiotics in the treatment of skin infections.

## Figures and Tables

**Table 1:** Molecular docking of GTN with the *P. aeruginosa* and *S. marcescens* QS targets.

QS Target	Compound	ΔG (kcal/mol)	RMSD (Å)	Interaction/Bond Type	Distance (Å)
*P. aeruginosa* receptor QscR	Co-crystallized ligand OHN	−10.08	1.48	ASP75: H-donor SER38: H-acceptor TYR58: H-acceptor TRP62: H-acceptor	2.82 2.89 2.89 3.21
	GTN	−7.06	1.066	TYR58: H-acceptor	2.99
*P. aeruginosa* receptor RhlR	Co-crystallized ligand HL4	−6.55	1.3	ASP81: H-donor TRP68: H-acceptor TYR64: H-acceptor SER135 H-acceptor TRP96: H-pi TRP96: H-pi	2.89 2.96 2.73 2.83 3.80 4.21
	GTN	−6.54	1.9	ASP81: H-donor TYR64: H-acceptor SER135: H-acceptor TRP68: H-acceptor	3.51 3.01 2.87 3.01
*P. aeruginosa* receptor LasR	Co-crystallized ligand 3M5	−10.63	1.00	ASP73: H-donor TRP60: H-acceptor TYR56: H-acceptor SER129: H-acceptor TRP88: H-pi	2.81 3.09 2.72 2.63 4.03
	GTN	−6.7	1.88	TYR56: H-acceptor SER129: H-acceptor ARG61: H-acceptor	2.82 2.94 2.94
*P. aeruginosa* receptor PqsR	Co-crystallized ligand NNQ	−6.5	1.3	ILE236: pi-H	3.78
	GTN	−6.3	1.15	GLN194: H-acceptor LEU197: H-acceptor	2.98 3.11
*P. aeruginosa* PqsA	Co-crystallized ligand 3UK	−10.5	1.3	GLY302: H-donor ASP382: H-donor ALA303: H-acceptor THR304: H-acceptor THR304: H-acceptor HIS308: H-pi GLY302: pi-H	2.60 2.79 3.28 2.74 3.00 3.71 3.74
	GTN	−5.88	1.3	GLY302: H-donor THR304: H-acceptor	3.23 3.01
	Co-crystallized ligand PEG	−3.71	0.667	GLU286: H-donor GLU286: H-donor	3.26 3.12
	GTN	−4.33	1.56	GLU286: H-donor	3.34
*P. aeruginosa* LasI-type	GTN	−4.85	1.6	ASN108: H-acceptor	3.03
*S. marcescens* receptor SmaR	Co-crystallized ligand LAE	−9.55	1.13	ASP70: H-donor TRP57: H-acceptor TYR53: H-acceptor	2.96 2.98 2.80
	GTN	−7.1	1.1	ASP70: H-donor TRP57: H-acceptor	3.57 3.18

## Data Availability

Not applicable.
